# Compatibility of the Predatory Beetle, *Delphastus catalinae,* with an Entomopathogenic Fungus, *Cordyceps fumosorosea,* for Biocontrol of Invasive Pepper Whitefly, *Aleurothrixus trachoides,* in Florida

**DOI:** 10.3390/insects11090590

**Published:** 2020-09-01

**Authors:** Pasco B. Avery, Vivek Kumar, Antonio Francis, Cindy L. McKenzie, Lance S. Osborne

**Affiliations:** 1 Indian River Research and Education Center, Institute of Food and Agricultural Sciences (IFAS), University of Florida, 2199 South Rock Road, Fort Pierce, FL 34945, USA; pbavery@ufl.edu; 2 IFAS, Mid-Florida Research and Education Center, University of Florida, 2725 S. Binion Road, Apopka, FL 32703, USA; lsosborn@ufl.edu; 3 Florida Department of Agriculture and Consumer Services, UF/IFAS Mid-Florida Research and Education Center Division of Plant Industry, 915 10th Street E, Palmetto, FL 34221, USA; Antonio.Francis@freshfromflorida.com; 4 Horticultural Research Laboratory, Subtropical Insect Research Unit, USDA-ARS, U.S., 2001 South Rock Road, Fort Pierce, FL 34945, USA; cindy.mckenzie@usda.gov

**Keywords:** biological control, ladybird beetle, insect-pathogenic fungus, multi-trophic interactions, ornamental pepper, *Isaria fumosorosea*

## Abstract

**Simple Summary:**

The solanum whitefly, *Aleurothrixus trachoides*, is a polyphagous pest known to attack > 70 crops worldwide. Endemic to the Neotropical region, in the past few years, it has emerged as a significant pest of several horticultural crops including pepper in the United States. To develop an eco-friendly sustainable management strategy for this pest, in this study, we evaluated the efficacy and compatibility of two commercially available biological control agents, predatory beetle *Delphastus catalinae* (*Dc*) and entomopathogenic fungi *Cordyceps fumosorosea* (*Cfr*) under laboratory conditions. Results showed both *Cfr*, treatments (alone or in combination with *Dc*) were efficacious, and *Cfr* did not negatively impact the beetle longevity. This study’s outcome is important not only for organic horticultural growers, but also for conventional growers as it offers a low-risk alternative tool for solanum whitefly integrated pest management in Florida and other affected regions.

**Abstract:**

*Aleurothrixus* (formerly known as *Aleurotrachelus*) *trachoides* Back (Hemiptera: Aleyrodidae), commonly known as pepper or solanum whitefly, is a new emerging whitefly pest of several horticultural crops in the United States. During the preliminary survey for pepper whitefly infestation in Florida, a whitefly-specific predatory beetle *Delphastus catalinae* Horn (Coleoptera: Coccinellidae) was observed associated with this pest in the natural ecosystem. The current study was undertaken to determine the efficacy of this naturally occurring predator, *D. catalinae*, and an entomopathogenic fungus, *Cordyceps* (formerly known as *Isaria*) *fumosorosea* (*Cfr*) (Wize) (Hypocreales: Cordycipitaceae), alone or in combination, under controlled laboratory conditions. Whitefly mortality for *Cfr* (88%), beetle (100%) and *Cfr* + beetle (100%) treatments were similar and significantly higher compared to control. In the combination treatment, *Cfr* did not impact *D. catalinae* longevity and daily food intake, indicating a neutral interaction between the two treatments. Results showed that both of these natural biological control agents could potentially offer an effective alternative in the battle against invasive whiteflies such as pepper whitefly in U.S. horticulture production, either as a stand-alone strategy or in an integrated approach. Although these findings are promising, compatibility of the two treatments needs to be evaluated further under greenhouse and semi-field conditions before recommending to commercial growers.

## 1. Introduction

Solanum or pepper whitefly, *Aleurothrixus trachoides* Back (Hemiptera: Aleyrodidae), is an emerging pest of Neotropical origin that has continued to spread and cause damage in some areas of Florida [1]. This whitefly has been in the United States for several decades as an occasional pest of pepper but was never a major economic concern [1]. Detection records from 1985 to 2015 showed that *A. trachoides* had been reported in 16 counties, with 45% of the records belonging to Miami-Dade County [2]. It has also been confirmed in California, Hawaii, Louisiana, Texas, parts of Africa, and the Pacific, and recently, in Karnataka, India [3]. *Aleurothrixus *trachoides** is a polyphagous pest, known to feed on > 70 different crops, with a preference for Solanaceae and Convolvulaceae [3]. List of preferred hosts include *Apium graveolens* L. (celery)*, Capsicum annuum* L. (sweet pepper)*, Capsicum frutescens* L. (chilli pepper), *Ipomoea batatas* (L.) Lam. (sweet potato)*, Nicotiana tabacum* L. (tobacco)*, Persea americana* Mill. (avocado)*, Rosa* sp. (rose), *Solanum lycopersicum* L. (tomato), and *Solanum melongena* L. (eggplant). Most of the host records in Florida are from pepper, sweet potato, *Cocos nucifera* (coconut palm), and *Solanum lycopersicum* L. (tomato) [2]. Besides, *Duranta erecta* L. (pigeon berry), which is commonly grown as a hedge in the Florida landscape, is severely affected by this pest [1]. Whitefly feeding damage is inflicted by removal of plant sap, increasing overall stress to the affected plant, with a subsequent buildup of wax, honeydew, and sooty mold on the plant foliage. Losses from heavy infestations may lead to stunted growth, lower fruit yield [3], and in some extreme cases, plant death. In addition, recently Chandrashekar et al. [4] discovered that this whitefly can transmit a begomovirus, Durant leaf curl virus, from *Durant* spp. to potato, tomato, and pepper plants which will have a great impact on solanaceous vegetable cultivation worldwide. 

Sparse biological information is available to develop effective management practices for *A. trachoides* because it is a relatively recent pest of economic relevance in Florida. However, the integration of fungal entomopathogens into existing pest control practices might provide an environmentally friendly alternative to traditional approaches. Past studies have shown that fungal entomopathogens are critical regulatory factors in managing arthropod pest populations [5,6,7]. These biological control agents are also safe for non-target organisms and are compatible with other natural enemies such as predators and parasitoids [8,9,10]. Among these fungal entomopathogens, *Cordyceps* (=*Isaria*) *fumosorosea* (Wize) Kepler, B. Shrestha & Spatafora (Hypocreales: Cordycipitaceae) is regularly used in greenhouse and nursery production systems where it has proven to be efficacious against various insect pests and can tolerate climatic conditions in the southern United States [11]. This entomopathogenic fungus, is available as a commercial product (Ancora^®^ = PFR-97^®^ 20% WDG) for control of some arthropod pests [12]. 

Based on the Universal Chalcidoidea Database (Natural History Museum, London, UK), *Encarsia brasiliensis* Hempel, *Encarsia cubensis* Gahan, *Encarsia formosa* Gahan, *Encarsia nigricephala* Dozier, *Encarsia pergandiella* Howard, *Encarsia tabacivora* Viggiani, and *Eretmocerus gracilis* Rose have been observed to parasitize this species; however, their use as effective biocontrol agents of *Aleurotrachelus trachoides* is still a subject of investigation [1]. During the recent surveys for *A. trachoides* infestations in Florida, we found two predatory beetles belonging to the genus *Delphastus* Casey (Coleoptera: Coccinellidae) associated with this pest in the urban landscape. Beetles of this genus are minute in size and are whitefly-specific predators. Three beetle species of this genus, *Delphastus catalinae* (Horn), *D. pallidus* LeConte, and *D. pussilus* LeConte are considered native to the Americas and have a wide distribution in the Florida landscape [13,14,15,16,17,18]. Among these beetle species, *D. catalinae* has received considerable attention in the past decade as a potential biocontrol agent of multiple whitefly species, including *Trialeurodes vaporariorum* Westwood, *Trialeurodes abutiloneus* Haldeman, *A. floccosus* Maskell, and *Dialeurodes* spp. as well as *Bemisia tabaci* [17,19,20,21]. Furthermore, Hoelmer and Pickett [16] suggested that due to the uncertainly on the taxonomic status of the of *Delphastus* spp. in the commercial insectary, most of the previous interaction studies between *B. tabaci* and *Delphastus pusillus* likely was *D. catalinae*. However, there is a lack of information on the feeding behavior of *D. catalinae* on *A. trachoides*. 

The main objectives of this study were to evaluate the efficacy and compatibility of the entomopathogenic fungus, *C. fumosorosea* with the lady beetle, *D. catalinae* alone and in combination for suppressing infestations of *A. trachoides* on ornamental pepper plants. The sub-objectives of these studies were (1) to determine laboratory and potential greenhouse efficacy and compatibility of *C. fumosorosea* alone and in combination with the predatory beetle for population suppression of the pepper whitefly and (2) to assess the potential negative non-target effect of the entomopathogenic fungus on the predatory beetle egg consumption rate and survival.

## 2. Materials and Methods 

### 2.1. Plants

The Red Missile plant cultivar of *Capsicum annuum* (L.) ornamental pepper (OP) were grown at the ARS Horticultural Laboratory Research Laboratory greenhouse in Fort Pierce, FL as described in Avery et al. [22]. Potted plants (75 days after planting) were transported to the Hayslip Biological Control and Containment Laboratory at the University of Florida, in Fort Pierce, FL to be used in the experiments.

### 2.2. Insects

The pepper whitefly colony was established using the following technique: OP Red Missile potted plant with 8–10 healthy similar-sized leaves was placed inside a Dacron chiffon (24 × 24 × 24 inches; 60.9 × 60.9 × 60.9 cm) collapsible cage (BioQuip Products, Rancho Dominguez, CA, USA) containing a pepper plant heavily infested with whitefly adults for 48 h to allow the whitefly to oviposit on the OP leaves. After this duration, the OP plant was removed from the cage and the remaining adult whiteflies found on the leaves were also removed. Some OP leaves containing eggs were used immediately in the egg consumption experiments. The other OP plants needed for experiments involving the consumption of whitefly 1st instars were placed in cages in the greenhouse for an additional 6 days. Predatory adult beetles of *D. catalinae* were purchased as Delphibug™ (Koppert Biological Systems, Howell, MI, USA) and used directly as obtained in the dispenser container. Adult beetles’ sexes were unknown, but ages were similar. Beetles prior to any experiment, were starved for 24 h and allowed to feed only on a moistened water wick.

### 2.3. Fungus

The commercially-available formulated fungal biopesticide, PFR-97 20% WDG (wettable dispersible granule) containing *C. fumosorosea* (*Cfr*) blastospores was prepared as follows: To produce the label rate of 10^6^ blastospores mL^−1^, 0.1 g of the PFR-97 powder was added to a 150 mL glass beaker with 100 mL of sterile distilled water, covered with aluminum foil and mixed for 30 min using a magnetic stirring bar. After this duration, the *Cfr* suspension was allowed an additional 30 min for the inert material to precipitate out of the suspension, leaving the blastospores suspended in the supernatant. Spore suspension of 10^6^ blastospores mL^−1^ in the supernatant was confirmed using an INCYTO C-Chip™ Improved Neubauer hemocytometer (INCYTO Co., Ltd., Chungcheongnam-do, Korea) viewed under a Leica DM500 compound light microscope (Leica Microsystems Inc., Buffalo Grove, IL, USA) at 400×. Percentage viability of the fungal product was determined by dividing the number of colony-forming units formed on the potato dextrose agar plates with that expected based on the label, times 100 [23]. The viability of the blastospore formulation was 100% at the time of testing.

### 2.4. Spray Protocol and Deposition of Blastospores

The spray platform consisted of an orange plastic cafeteria food tray (US Plastic Corporation^®^, Lima, OH, USA) with the bottom lined with brown paper towels. Detached leaves or leaf sections cut out of the leaf of ornamental pepper plants containing whitefly eggs were placed on top of the paper toweling. In addition, plastic coverslips were randomly dispersed among the leaves or leaf sections on the spray platform to determine the deposition of blastospores mm^−2^ area. Separate platforms containing the leaves or leaf sections were placed inside a spray chamber and then sprayed with either water only or with the PFR-97 suspension using a Nalgene^®^ aerosol hand pump sprayer (Nalge Nunc International, Rochester, NY, USA). Leaves or leaf sections were sprayed to the point of runoff and then allowed to air dry before being placed in the bioassay arenas. Mean spore deposition, determined using a similar protocol to that of Avery et al. [24] was 157 ± 35.8 blastospores mm^−2^ for all the experiments.

### 2.5. Laboratory Protocol for Determining Efficacy of Treatments on the Mortality of Whitefly Populations 

**Figure 1:**
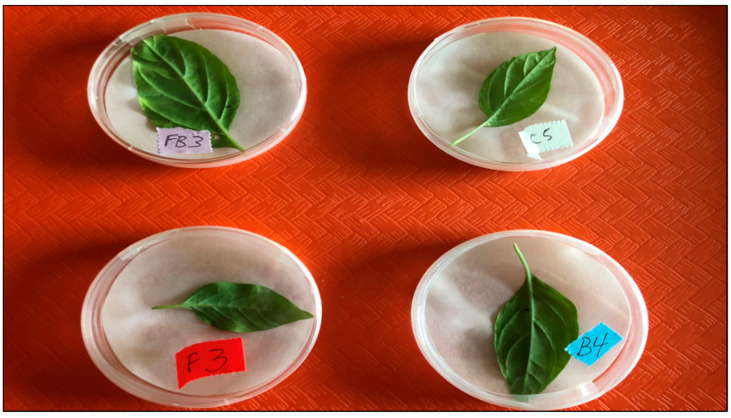
Bioassay experimental arena showing placement of treated detached leaves over moistened filter paper.

Predatory adult beetles were used in replicate experiments directly as obtained in the dispenser container. Laboratory bioassay arenas for these studies consisted of a Petri™ dish (55 mm diameter) with screen placed over a hole created in the top cover which provided ventilation. Each dish contained a moistened filter paper (55 mm) placed in the bottom of the dish and a Red Missile OP leaf or leaf section containing pepper whitefly 1st instars was placed on top of the filter paper to be exposed to the beetle. Treatments consisted of a detached leaf totaling 50 whitefly 1st instars (10 per dish): (1) sprayed with water only and allowed to air dry; (2) sprayed with water and allowed to air dry, and then a single beetle starved for 24 h was released in the arena; (3) sprayed with the *Cfr* fungal product PFR-97 at a rate of 10^6^ blastospores mL^−1^ to the point of runoff and allowed to air dry; and (4) sprayed with the *Cfr* fungal product PFR-97 at a rate of 10^6^ blastospores mL^−1^ to the point of runoff and allowed to air dry, then a single beetle starved for 24 h was released in the arena (Figure 1). Dishes with un-vented top covers were sealed with Parafilm™ (Bemis Co., Neenah, WI, USA) for 24 h to allow the fungus time to germinate and potentially infect the whitefly eggs and 1st instars. 

After this time, vented top covers were placed on top of the dish bottoms and the bioassay arenas were randomized with each treatment in a row on a single tray; there were 5 rows per tray. Trays were transferred to a growth chamber set at 25 °C with approximately 55 ± 4.2% RH under a 14:10 h photoperiod. After each daily assessment, filter papers were moistened daily as needed to maintain a constant relative humidity inside the arenas, sealed with Parafilm and returned to the growth chamber as above. Nymphs were assessed daily using a Leica EZ4 HD binocular dissecting scope (Leica MicroSystems, Buffalo Grove, IL, USA), at 40× until all 1st instars were consumed and/or molted over a 7-day period. There were 5 replicate arenas per treatment and the experiment was conducted twice for a total of 10 replicate arenas per treatment. Dead beetles were removed from the arenas daily, surface sterilized, placed in a moistened filter paper chamber, sealed with Parafilm, and then transferred to the growth chamber. This protocol allowed the fungus to mycose on the beetle and verify if death was primarily due to *Cfr* infection. All the nymphs in the combination treatment #4 were consumed by the beetles; therefore, the assessment for mycosis due to *Cfr* infection was not necessary. 

### 2.6. Longevity of Adult Beetles after Exposure to Entomopathogenic Fungus

The longevity of beetles in treatment 2 (described above) was compared with those in treatment 4 (described above) to assess the potential infection and negative non-target effect of the entomopathogenic fungus; however, in this study beetles consumed whitefly eggs. Live beetles in treatments were removed after their consumption period (24 h) of 50 treated or untreated eggs. After this duration, the beetles were placed in a new arena and given fresh untreated whitefly eggs every 3 days for 21 days or until death. Eggs were viewed and counted using a Leica EZ4 HD binocular dissecting scope (Leica MicroSystems, Buffalo Grove, IL, USA) at 40×. Dead beetles were removed from the arenas daily, surface sterilized, placed in a moistened filter paper chamber, sealed with Parafilm, and then transferred to the growth chamber under the same conditions as described above. This protocol allowed the fungus to mycose on the insect and verify if death was primarily due to *Cfr* infection. There were 5 replicate arenas per treatment and the experiment was conducted two times for a total of 10 replicate arenas per treatment. 

### 2.7. Statistical Analysis

To determine if percent mortality of beetles was significantly different among treatments in the laboratory bioassay study, data were analyzed using generalized linear model with the SAS^®^ procedure GLIMMIX, with the model distribution statement specified as binomial. Differences among treatment means were separated using Fisher’s LSD test (α = 0.05). The number of whitefly eggs, 1st instar nymphs consumed, and daily consumption rate of the adult beetles were compared using a Student’s *t*-test (*p* < 0.05). Untransformed data were presented in the graphs. Survival times of adult beetles were compared for significant differences between treatments using the Kaplan—Meier survival analysis (*p* < 0.05) followed by a log-rank test (SAS JMP Pro 13 for Windows). The Student’s *t*-test statistical analysis was conducted using the PROC TTEST program. All statistical analyses except survival of the beetle were conducted using SAS 9.4 for WINDOWS 2012 (SAS Institute, Inc., Cary, NC, USA).

## 3. Results

### 3.1. Efficacy of Treatments on the Mortality of Whitefly Populations

**Figure 2:**
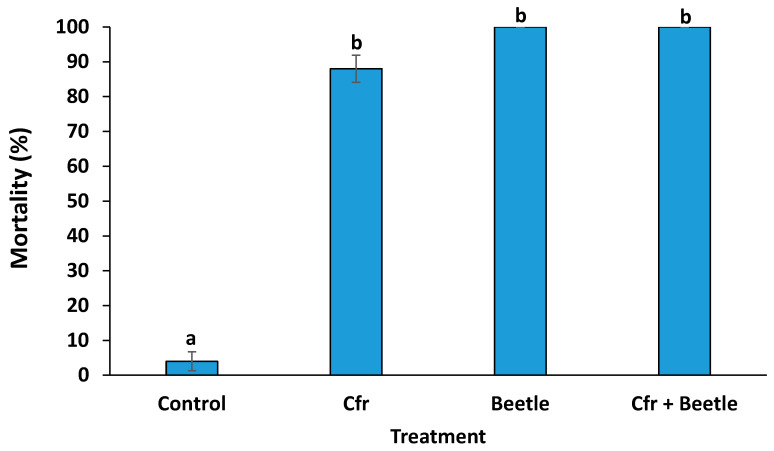
Mean mortality (±SEM) of *Aleurothrixus trachoides* nymphs after Red Missile ornamental pepper plant leaves were sprayed with: water only (control), and allowed to air dry; *Cordyceps fumosorosea* (Cfr) only; water only, and then one adult *Delphastus catalinae* (beetle) was released; and *C. fumosorosea,* and then one adult *D. catalinae* beetle was released (Cfr + beetle). Letters above the bars (±SEM) that are the same are not significantly different (Fisher’s LSD test, *p* < 0.05).

Data for both trials were combined and analyzed together after it was determined that trial x treatment interaction was not significant (*F* = 0.30; df = 3, 24; *p* = 0.8216). Mortality ± SEM of the whitefly nymphs for *Cfr* (88%), beetle (100%) and *Cfr* + beetle (100%) treatments were similar and significantly higher (*F* = 3.57; df = 3, 36; *p* = 0.0233) compared to control (4%) (Figure 2). 

### 3.2. Consumption of Fungus-Contaminated Whitefly Eggs by Beetles in No-Choice Arenas 

**Figure 3:**
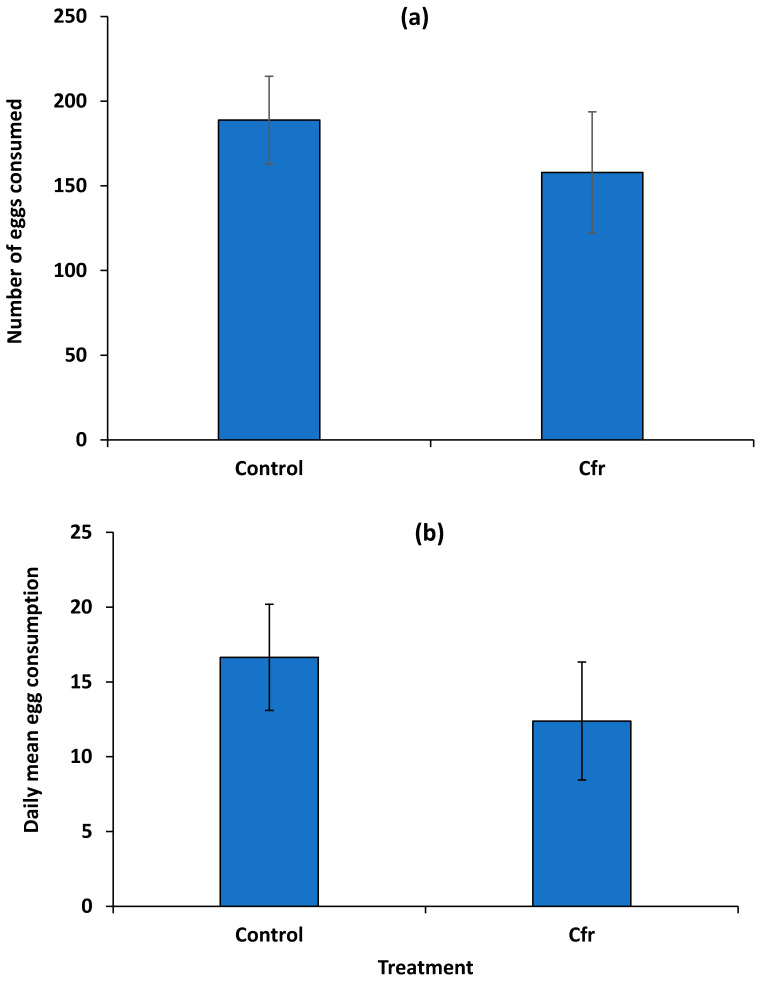
Total number (±SEM) of *Aleurothrixus trachoides* eggs consumed (**a**) and daily mean (±SEM) consumption rate (**b**) by an adult *Delphastus catalinae* beetle after being treated with either *Cordyceps fumosorosea* (Cfr) or water (control) on leaves of red missile ornamental plants for 24 h. The daily mean egg consumption or consumption rate by the adult beetle was not significantly different between treatments (Student’s *t*-test, *p* > 0.05).

Consumption of eggs sprayed with *Cfr* (157 ± 35.8) in the no-choice arenas by the beetles was not significantly different (*t* = −0.70; df = 1; *p* = 0.4917) compared to the control (188 ± 25.9) over time (Figure 3a). In addition, the daily mean consumption rate of whitefly eggs by the adult beetles over time was not significantly different (*t* = −0.80; df = 1; *p* = 0.4323) between the *Cfr*-treated eggs (12 ± 3.9) and the control (17 ± 3.6) over time (Figure 3b).

### 3.3. Longevity of Beetles after Exposure to Fungus-Contaminated Whitefly Eggs

**Figure 4:**
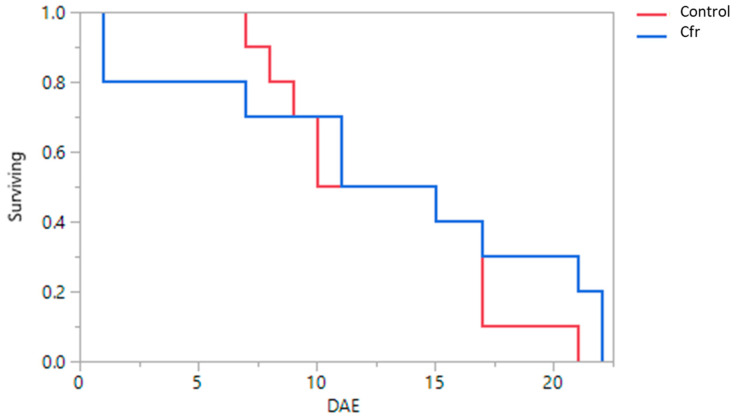
Survival curves of adult *Delphastus catalinae* beetle days after exposure (DAE) to *Aleurothrixus trachoides* eggs treated with either *Cordyceps fumosorosea* (Cfr) or water (control) on leaves of Red Missile ornamental plants for 24 h. Fresh untreated eggs were provided to the beetles every 3 days after the 24 h exposure period for 21 days. The median survival times of the adult beetles were not significantly different between treatments (log-rank test, *p* > 0.05).

Using the Kaplan—Meier survival analysis, the median survival time of beetles after exposure of eggs treated with *Cfr* compared to control was not significantly different (log-rank test: *χ*^2^ = 0.5914; df = 1; *p* = 0.4419; Figure 4). The daily mean survival time ± SEM of the beetles after exposure to whitefly eggs contaminated with *Cfr* (13 ± 2.5) did not differ significantly (*t* = −0.10; df = 1; *p* = 0.9204) compared to the control (13 ± 1.5). 

## 4. Discussion

The current study demonstrated the potential utility of naturally occurring biological control agents in managing invasive whitefly pests; however, this study was conducted under laboratory superficial conditions within a closed system. There was 100% whitefly nymphal mortality observed in *D. catalinae* treatments alone, and with *Cfr,* indicating the importance of this predator in implementing control measures for pepper whitefly. In Florida urban landscapes, the presence of this beetle along with the pepper whitefly indicated an association between two arthropods, and this prey–predator interaction has been confirmed from this study. Along with their whitefly specificity, feeding potential, and reproductive rate, the ability of this beetle genus to avoid parasitized whitefly stages [17,25] is a boon for the establishment of a biological control program in an urban setting or in organic nurseries with limited resources available to manage whitefly populations. Moreover, *D. catalinae* is a generalist predator and shows a density-dependent feeding response in relation to the fluctuation in the pest populations, which ensures the long-term presence of this predator in areas of pest infestation [26].

High pepper whitefly mortality (~88%) in the *Cfr*-alone treatment demonstrated that *Cfr* could be an important tool to combat this invasive whitefly. Results of the current study align with several studies around the globe where *Cfr* was found efficacious against multiple whitefly species [11,24,27,28,29,30,31,32,33,34,35]. Given the favorable conditions (temperature 24–30 °C + high humidity) [28], the unique mode of action of *Cfr*, either by cuticular invasion or ingestion *per os*, can lead to the death of the organism. It can kill the pest by mechanical damage to the tissue, depletion of nutritional content, and/or production and spread of toxins [36,37]. In the past, researchers isolated *Cfr* from a mycosed hemipteran pest such as *B. tabaci,* and *Diaphorina citri* Kuwayama, indicating the prevalence of this insect mycopathogen in the Florida production systems [27,38]. Considering the importance of low-risk pesticides being applied in regions with infestations of the pepper whitefly, *Cfr* offers an excellent IPM tool. Our results demonstrate the potential of this natural enzootic as a microbial candidate, and, thus *Cfr* should be tested against pepper whitefly under various production system parameters (controlled greenhouse, screen house, and open field) as well as variable pest pressure scenarios.

There are three kinds of biotic interactions—antagonistic, synergistic, or neutral, which can be expected when two different organisms are combined. In the current study, we observed that there was a 100% mortality in the beetle alone and beetle + *Cfr* treatment. Although, no adverse interaction between the two treatments was apparent, their additive response could not be assessed due to the low initial count of whitefly used in the bioassays. We assume if a higher population of pepper whitefly was used in this study, a clear differentiation in the activity of the three treatments could be observed. Our results indicate that the whitefly eggs or nymphal stages sprayed with *Cfr* did not impact the daily egg consumption rate or longevity of *D. catalinae*. Barahona et al. [12] also observed that after the ladybird beetle, *Thalassa montezumae* Mulsant consumed green croton scales sprayed with *Cfr* on croton plants, the survival rate was similar to those feeding on untreated scale insects. In a recent unpublished study, the metallic blue beetle, *Curinus coeruleus* Mulsant consumed Florida red scale insects sprayed with *Cfr* at the same rate as those not sprayed with the entomopathogenic fungus [39]. In our study, the predator was only exposed to the residue of the fungus and not to the spray directly as were the whitefly prey. Therefore, based on the results of this study we can suggest in an IPM scenario that when both *D. catalinae* and *Cfr* are used together, it is best to spray the fungus first, and allow it to dry prior to releasing the predator. However, in a greenhouse study, Alma et al. [9] observed that the combination of a mirid predator after being sprayed with *Cfr* while feeding on greenhouse whitefly nymphs found on tomato plants, that the efficacy of the predator was not negatively affected compared to the control. In corroboration with our laboratory study, Poprawski et al. [40] observed that the adult ladybird beetles (*Serangium parcesetosum* Sicard) sprayed with *Cfr* consumed whitefly nymphs (*Bemisia* sp.) at a rate similar to the controls and survivorship of the adult beetles did not significantly differ from the controls. In further support of our results, Zhou et al. [41] in studying the effects of *Cfr* applied directly on the adult ladybird beetles (*Axinoscymnus cardilobus* Pang and Ren) under laboratory conditions found that the fecundity, longevity, egg viability, and life table parameters of the females were not significantly different compared the control. Therefore, when both agents are used in combination, it seems that the use of the fungal entomopathogen, *Cfr* is compatible and can be applied in combination with a variety of ladybird beetle predators for managing arthropod pests.

## 5. Conclusions

This study compared the potential compatibility and utility of a natural predator and enzootic insect fungal pathogen when used alone or in combination against a new invasive whitefly pest in Florida under controlled laboratory conditions. Both *D. catalinae* and *Cfr* were efficacious against the pepper whitefly, and *Cfr* as a residue on the whitefly life stages did not impact the predator’s longevity. Considering the importance of low-risk alternative management practices needed today, this study offers a few new potential tools for the IPM of pepper whitefly in Florida and other affected regions. However, this hypothesis needs to be confirmed using larger-scale trials under greenhouse and field conditions. Therefore, future studies will focus on evaluating the compatibility of the fungal entomopathogen *Cfr* alone and in combination with the ladybird beetle under greenhouse and field conditions to confirm the laboratory results.
